# metaConvert: an automatic suite for estimation of 11 different effect size measures and flexible conversion across them

**DOI:** 10.1017/rsm.2025.11

**Published:** 2025-04-02

**Authors:** Corentin J. Gosling, Samuele Cortese, Marco Solmi, Belen Haza, Eduard Vieta, Richard Delorme, Paolo Fusar-Poli, Joaquim Radua

**Affiliations:** 1 Université Paris Nanterre, Laboratoire DysCo, Nanterre, France; 2 Department of Child and Adolescent Psychiatry, Robert Debré Hospital, APHP, Paris, France; 3 Developmental EPI (Evidence synthesis, Prediction, Implementation) Lab, Centre for Innovation in Mental Health, School of Psychology, Faculty of Environmental and Life Sciences, University of Southampton, Southampton, UK; 4 Clinical and Experimental Sciences (CNS and Psychiatry), Faculty of Medicine, University of Southampton, Southampton, UK; 5 Hampshire and Isle of Wight Healthcare NHS Foundation Trust, Southampton, UK; 6 Hassenfeld Children’s Hospital at NYU Langone, New York University Child Study Center, New York City, NY, USA; 7 DiMePRe-J-Department of Precision and Regenerative Medicine-Jonic Area, University of Bari “Aldo Moro,” Bari, Italy; 8 Department of Psychiatry, University of Ottawa, Ottawa, ON, Canada; 9 Department of Mental Health, The Ottawa Hospital, Ottawa, ON, Canada; 10 School of Epidemiology and Public Health, Faculty of Medicine, University of Ottawa, Ottawa, ON, Canada; 11 Ottawa Hospital Research Institute (OHRI), Clinical Epidemiology Program, University of Ottawa, Ottawa, ON, Canada; 12 Department of Child and Adolescent Psychiatry, Charité Universitätsmedizin, Berlin, Germany; 13 Institut d’Investigacions Biomediques August Pi I Sunyer, University of Barcelona, Barcelona, Spain; 14 Centro de Investigacion Biomedica en Red de Salud Mental (CIBERSAM), Barcelona, Spain; 15 Department of Psychiatry and Psychology, Hospital Clinic, Institute of Neuroscience, University of Barcelona, Barcelona, Spain; 16 Université Paris Cité, Paris, France; 17 Department of Brain and Behavioral Sciences, University of Pavia, Pavia, Italy; 18 Early Psychosis: Interventions and Clinical-detection (EPIC) Lab, Department of Psychosis Studies, King’s College London, London, UK; 19 Outreach and Support in South-London (OASIS) service, South London and Maudsley (SLaM) NHS Foundation Trust, London, UK; 20 Department of Psychiatry and Psychotherapy, University Hospital, Ludwig-Maximilian-University (LMU), Munich, Germany

**Keywords:** effect size, meta-analysis, R, shiny app

## Abstract

A fundamental pillar of science is the estimation of the effect size of associations. However, this task is sometimes difficult and error-prone. To facilitate this process, the R package metaConvert automatically calculates and flexibly converts multiple effect size measures. It applies more than 120 formulas to convert any relevant input data into Cohen’s *d*, Hedges’ *g*, mean difference, odds ratio, risk ratio, incidence rate ratio, correlation coefficient, Fisher’s r-to-z transformed correlation coefficient, variability ratio, coefficient of variation ratio, or number needed to treat. Researchers unfamiliar with R can use this software through a browser-based graphical interface (https://metaconvert.org/). We hope this suite will help researchers in the life sciences and other disciplines estimate and convert effect sizes more easily and accurately.

## Highlights

### What is already known

Effect size estimation in quantitative evidence synthesis is a time-consuming and error-prone step.

### What is new

We propose a new R package that automatically calculates and flexibly converts multiple effect size measures. An open-access, browser-based application provides a graphical user interface to key features proposed in this R package.

### Potential impact for research synthesis methods readers

Readers will benefit from a new, user-friendly tool that will make the process of estimating effect sizes easier and more reliable. While these tools are primarily dedicated to researchers performing a quantitative evidence synthesis, they can be useful to any researcher interested in estimating an effect size.

## 1 Introduction

Over the past few decades, numerous guidelines have emphasized the need to quantify and report the magnitude of the effect studied, in addition to the simple assessment of statistical significance.^1^ The estimation of an effect size is also the cornerstone of meta-analyses, which have become an essential tool for assessing the strength and credibility of an effect in the life sciences.^2^ However, estimating an effect size, particularly in the context of meta-analyses, involves many challenging and error-prone steps. Extracting appropriate information from primary studies, properly calculating an effect size, and handling dependent effect sizes are all common sources of error in published meta-analyses.^3^ ^–^ ^7^

Several pieces of software allow estimating effect sizes from various types of input data, including commercial software (such as *Comprehensive Meta-analysis*),^8^ free software (such as the *esci* JAMOVI module*, or* RevMan),^9^ ^,^ ^10^ R packages (such as *esc, compute.es, metafor*),^11^ ^–^ ^13^ and free online calculators.^14^ However, estimating effect sizes for a meta-analysis with these tools is not always straightforward for distinct reasons (Supplementary Figure S1). *First*, many of the existing tools do not offer an automatic workflow. This requires users to sequentially estimate effect sizes from each type of input data (i.e., from each statistical indicator that allows the computation of an effect size). For example, if users have extracted four types of input data – such as means and standard deviations, ANOVA *F*-values, Student’s *t*-test *p*-values, and medians, ranges, and interquartile ranges – they must sequentially calculate effect sizes for each type. This increases the risk of error (and makes the process very time-consuming) in situations where studies report a large array of input data that enables the computation of an effect size. *Second*, many of the available pieces of software are not flexible. Users must gather very specific types of input data to estimate each effect size measure available in the software. For example, no software currently allows the estimation of an SMD from both the results of an ANCOVA model and the medians, ranges, plus interquartile ranges, while this need is commonly found in research practice. This forces users to master and switch between different software programs to access specific features. *Third*, no software currently proposes convenient solutions for automatically comparing estimates of the same effect computed from multiple types of input data. For example, when exploring the effect of an intervention (regardless of whether it is surgical, pharmacological, or psychosocial), it is common that the same report gives access to the means of the two groups at post-test, the mean differences in the change from baseline of the two groups, as well as the results of a model exploring the effect of the intervention but adjusting for covariates. In this perspective, a previous study has shown that the choice of such types of input data has a great influence on the effect size estimates (a median of SMD = |0.30|).^15^ Therefore, there is a need to develop and implement convenient features to easily visualize the consistency of effect sizes generated from different types of input data (but quantifying the same effect), and implement them in software.

Although less critical, other aspects reinforce the difficulties associated with estimating an effect size during evidence synthesis. A critical assumption of traditional meta-analytic models is that the effect sizes are independent. However, it is very common for meta-analytic datasets to include dependent effect sizes, for example, because a single study uses multiple measures of the same outcome, multiple time points, or multiple subgroups. Accounting for this dependency is often overlooked, even if it could lead to substantial biases in the pooled estimates.^16^ Providing convenient tools to handle dependency between effect size estimates is thus critical in evidence synthesis research. Moreover, many meta-analytic datasets contain errors at the data extraction stage.^5^ Therefore, major guidelines for systematic reviews and meta-analyses require data extraction to be completed in duplicate by independent pairs of data extractors.^17^ However, when working with large datasets, performing this comparison can become a challenging task. Users would thus benefit from facilities that assist in the comparison of multiple datasets, implemented directly in the software they use to estimate the effect sizes.

To overcome these limitations of existing tools, we have developed a new comprehensive suite, named metaConvert, available both as R package and an open-access browser-based graphical interface (https://metaconvert.org). metaConvert aims to facilitate the calculation and conversion between different effect size measures. More generally, this suite directly addresses the needs expressed by researchers conducting a meta-analysis,^18^ but can benefit any researcher who needs to estimate an effect size.

## 2 Methods

### 2.1 General overview of the metaConvert tools

**Figure 1:**
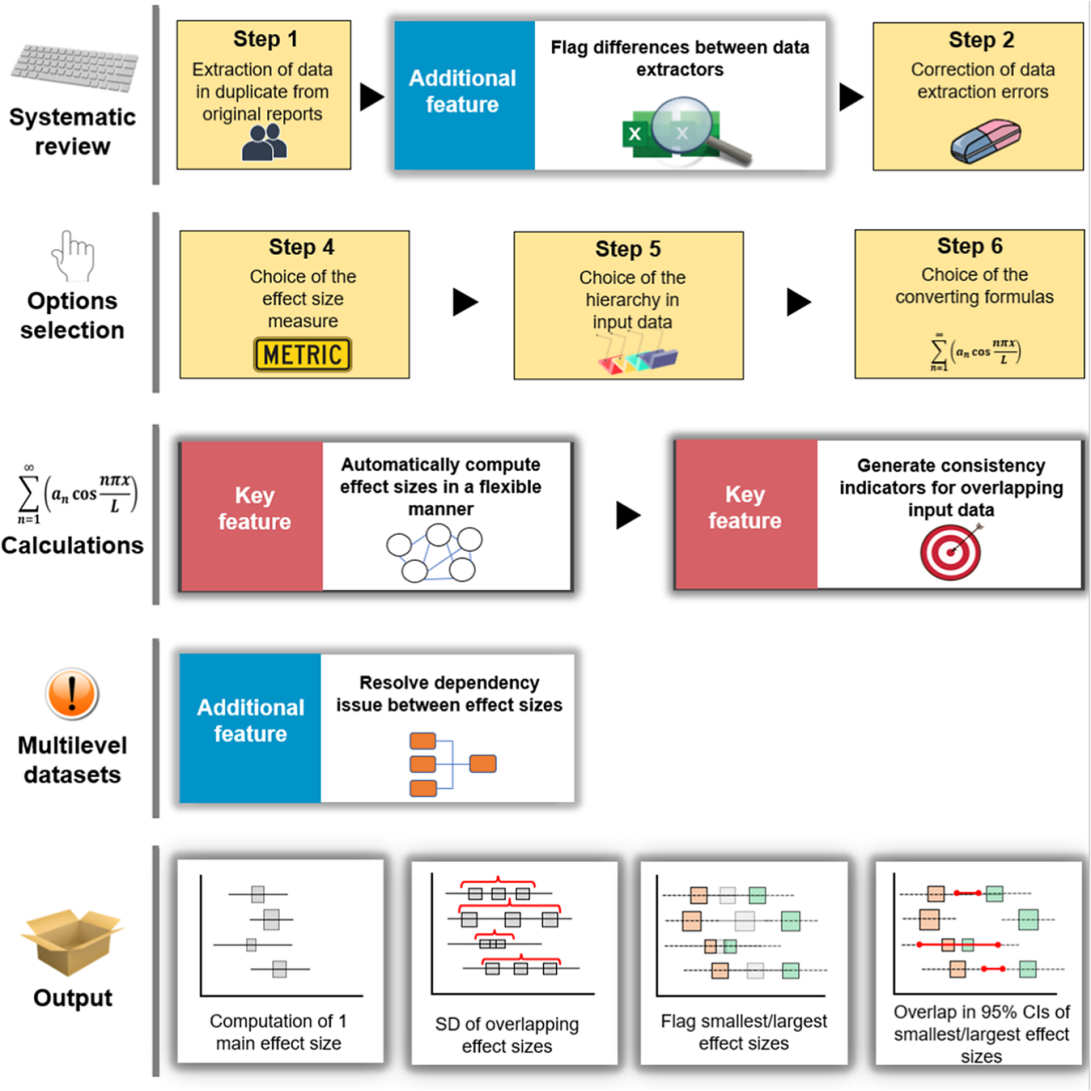
Visual representation of the automated workflow of the metaConvert tools in the framework of a systematic review with a meta-analysis. All boxes in yellow are completed by the users while others are automatically handled by the metaConvert tools. At the data extraction stage, the first additional feature assists users by helping identify differences between information extracted by two independent data extractors. At the calculations stage, the two key features allow users (i) to obtain effect sizes generated automatically from a wide range of input data and (ii) to easily determine whether overlapping input data yield consistent effect sizes. Last, after the effect size estimation, the third additional feature allows users to use standard meta-analytic models when there are dependencies between effect sizes, by aggregating the dependent values into one independent value.

The metaConvert tools are organized around three key features (Figure 1).

The first key feature implements an automatic and flexible effect size computation process, by relying on a core function that automatically calls more than 120 formulas estimating (or converting between) 11 effect size measures from various types of input data.

The second key feature is an innovative system designed to handle situations where several overlapping types of input data are present to estimate the same/a very similar estimand (i.e., situations where different statistical indicators are reported by the same study to estimate the same effect; e.g., a contingency table, an odds ratio value and 95% confidence interval [CI], and the proportions of events in two groups). This innovative feature enables users to (1) automatically calculate all effect sizes derived from all types of input data available and create a personalized selection process to retain only one of these effect sizes for the primary analysis, plus (2) to assess the consistency of effect sizes generated by the different types of input data.^i^

The additional features of the metaConvert tools are functions facilitating dataset comparisons between two data extractors, and facilitating the handling of dependent effect sizes.

### 2.2 Key feature: Automatically compute effect sizes in a flexible manner

**Figure 2:**
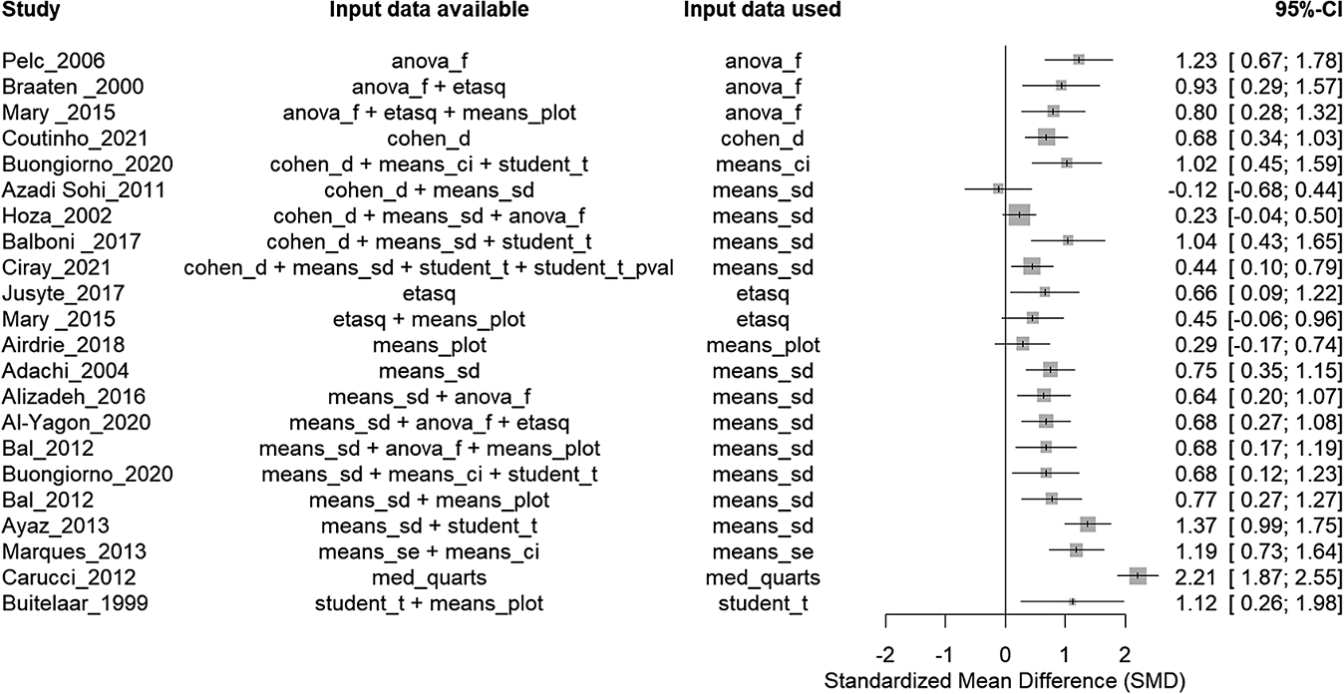
Example of plot showing, for each study, the main effect size, the input data used to generate it, and all the types of input data available to estimate the effect size measure. This plot can be generated directly from the output information generated by the convert_df() function and is automatically generated in the associated web-app.

The *convert_df()* function automatically generates effect size estimates, standard errors, and 95% CIs, directly from a well-formatted dataset (Figure 2). This function can estimate 11 effect size measures (Cohen’s d [D], Hedges’ g [G], mean difference [MD], odds ratio [OR], risk ratio [RR], incidence rate ratio [IRR], correlation coefficient [R], Fisher’s r-to-z transformed correlation coefficient [Z], variability ratio [VR], coefficient of variation ratio [CVR] and number needed to treat [NNT]). The function supports 127 formulas across 74 distinct input data combinations, with some combinations allowing multiple validated calculation methods. For example, because the conversion of an odds ratio to a correlation coefficient can be computed through several statistical approaches, the function implements several validated methods to ensure robust estimation across different contexts. All formulas are documented in the package documentation (https://cran.r-project.org/web/packages/metaConvert/metaConvert.pdf).

The key strength of this function over most other similar software programs dedicated to effect size computation lies (in addition to automation) in the flexible process employed. Users are only required to input a well-formatted dataset, and this function generates the desired effect size values, standard errors, and confidence intervals from any relevant input data stored in this dataset. The combination of automation and flexibility offers several advantages. First, it ensures a high reliability in calculating effect sizes across users. Second, it saves a substantial amount of time (by preventing users from building long scripts needed to estimate effect sizes from different types of input data). Third, as described in the next ‘key feature’ section, it enabled us to develop a new innovative system that can easily handle situations where users have access to multiple input data to quantify the same effect.

### 2.3 Key feature: Handling of overlapping input data

In instances where a dataset contains overlapping types of input data (i.e., several statistical indicators allowing to estimate effect sizes representative of the same or highly analogous estimand), the metaConvert tools automatically calculate all effect sizes derived from all available types of input data. By default, users can select a single “main” effect size from these generated estimates, simplifying their data analysis plan. The tools also evaluate the consistency among effect sizes derived from different input data types.

#### 2.3.1 Selection of one main effect size

Users can select a main effect size through three approaches:

1. Creating a custom hierarchy (e.g., prioritizing post-test means and standard deviations over baseline change scores when computing SMD)
2. Using pre-defined hierarchies for each effect size measure.
3. Selecting automatically the type of input data that produces either the smallest or largest effect size, enable sensitivity analyses to test the robustness of the results.

#### 2.3.2 Generate consistency indicators

**Figure 3:**
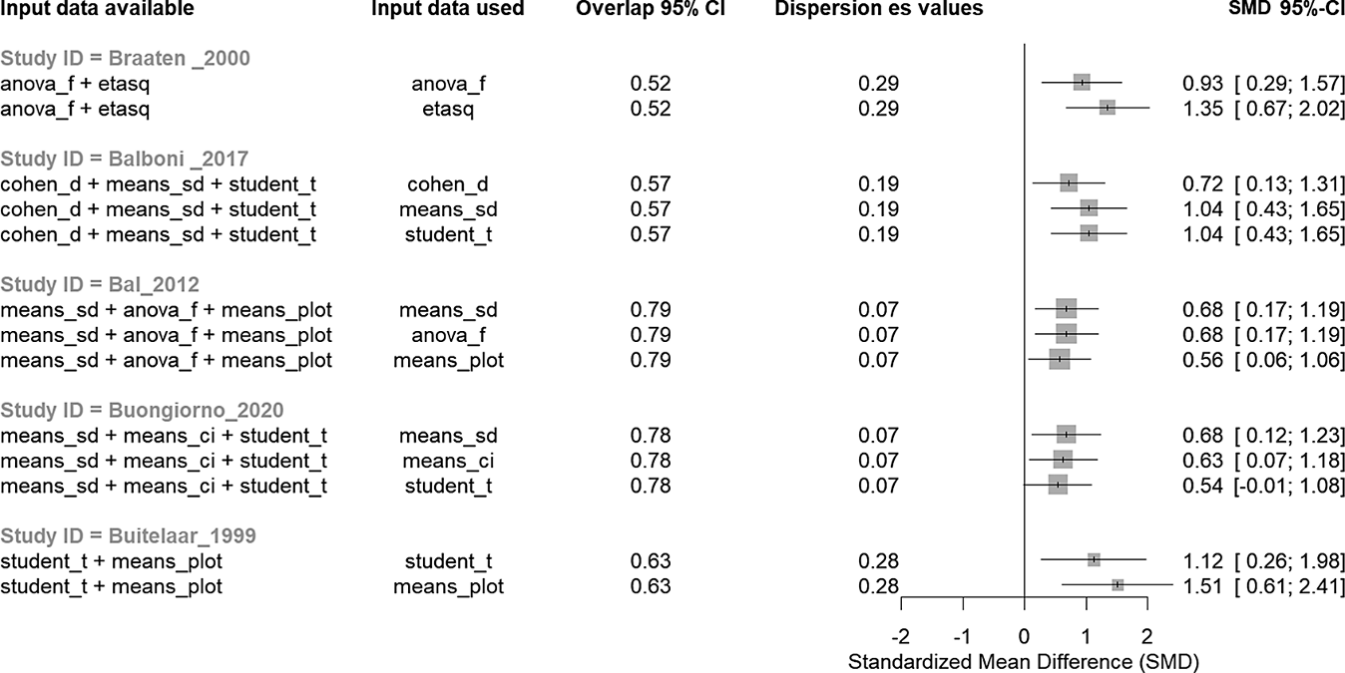
Example of plot showing, for some studies with overlapping data, the consistency in effect sizes depending on the type of input data used to estimate them, as well as consistency indicators. This plot can be generated directly from the output information generated by the convert_df() function.

The tools generate five key indicators to assess the consistency of effect sizes generated for estimands with overlapping input data: (i) the smallest effect size, (ii) the largest effect size, (iii) the difference between the smallest and largest effect size, (iv) the standard deviation of the effect size values, and (v) the percentage of overlap between the 95% CIs of the smallest and largest effect sizes. Identifying the smallest and largest effect sizes, as well as their difference, allows users to readily discern the types of input data that result in more extreme effect size values, and to evaluate the magnitude of their divergence. The standard deviation of effect size values provides a straightforward mean of understanding the dispersion of all effect sizes generated by the different types of input data. Furthermore, the percentage overlap of the 95% CI of the smallest and largest effect sizes allows users to gain insight not only into the differences in effect size values but also into the differences in effect size variances. These five indicators enable a clear visualization of the consistency of the effect sizes obtained from different input data (Figure 3).

#### 2.3.3 Implementation workflow

This approach improves upon current practice, which typically uses only one arbitrary type of input data, by (i) extracting all available types of input data, (ii) generating a main effect size from a preferred type of input data, and (iii) visualizing consistency across different types of input data types.

#### 2.3.4 Important considerations

While this system enhances transparency in the selection of input data, it does not limit the degree of freedom in decision-making. Therefore, it is crucial that meta-analysts outline in their protocol how they will handle overlapping input data types when planning their data analysis strategy. This should involve either defining a clear hierarchy for data selection or explicitly stating their reliance on the pre-established hierarchies provided by metaConvert. Failing to do so may result in selective data extraction. To aid in this process, users can refer to the proposed hierarchies for different effect measures, and, if needed, adjust them using interactive tables that display all available input data for estimating each effect size measure: https://metaconvert.org/input.html.

#### 2.3.5 Error preventions

Finally, we believe this feature will help reduce the risk of data extraction errors. For instance, if data extractors forget to reverse the direction of an effect size estimated from a specific input, metaConvert will flag the discrepancy by identifying effect sizes of equal magnitude but opposite directions. Another common error, such as a typo during data extraction (e.g., entering an OR value of 3.1 instead of 2.1), would be caught by the software, as the noticeable difference between the smallest and largest effect sizes would alert the data analyst to potential mistakes.

### 2.4 Additional feature: Flag differences between data extractors

**Figure 4:**
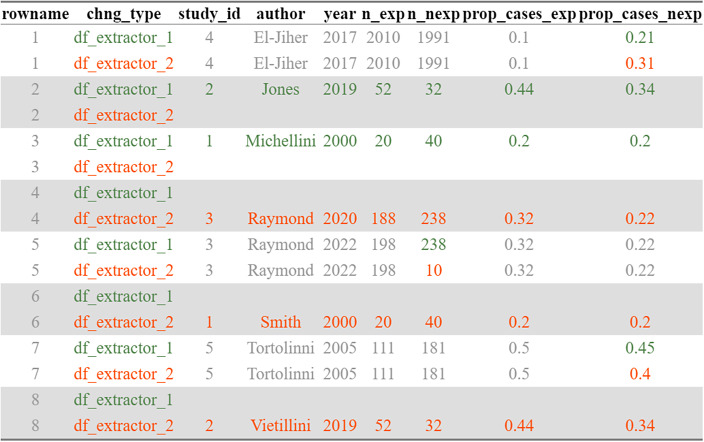
Exact image returned by the compare_df() function, highlighting differences between two datasets.

The *compare_df()* function simplifies the comparison of datasets by automatically identifying the information that differs between two datasets. This function is a wrapper around various functions from the R compareDF package,^19^ tailored specifically to the needs of researchers conducting meta-analyses (Figure 4).

### 2.5 Additional feature: resolve dependency issue between effect sizes

The *aggregate_df()* function proposed in the metaConvert tools implements three formulas that remove dependency between several effect sizes by aggregating them into one independent effect size.^20^ With these formulas, the metaConvert tools allow users to handle situations where a unique study includes several dependent effect sizes because (i) participants have completed multiple measurements of the same outcome (such as by using several measures of the same outcome), (ii) participants have completed the same outcome at different time-points, or (iii) multiple independent subgroups have completed the same outcome (e.g., boys and girls).

## 3 Results

We present here an example of the use of each feature of the metaConvert tools. All the steps completed using the R package are reproduced using the web-app (Supplementary Figure S2). It is crucial to note that the automatic calculations proposed in the metaConvert tools necessitate that users format their datasets in accordance with fixed rules. Prior to the presentation of the examples, we will provide a concise overview of the facilities that we have developed to obtain the required dataset formatting.

### 3.1 Example: format the dataset

#### 3.1.1 Using the R software

In R, users have the option of generating a dataset comprising the requisite columns (and a description of the information expected in each of these columns) for estimating the various effect measures proposed in metaConvert. The output can be a “data.frame” object, which can be viewed directly in the R console/viewer, or a regular file (such as .txt, .csv or .xlsx).

**Figure:**
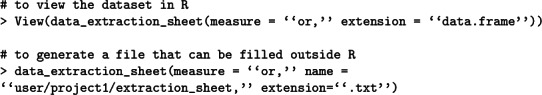

#### 3.1.2 Using the metaConvert website

The metaConvert website offers an interactive tool that allows users to create customized data extraction sheets. To utilize this tool, users must first indicate the type of input data from which they would like to estimate their effect sizes. Once this information has been entered, the tool will generate a dataset that adheres to the prescribed formatting conventions (https://metaconvert.org/input.html).

### 3.2 Example: automatically compute effect sizes in a flexible manner using R

#### 3.2.1 Automatic computation of the main effect size

To automatically compute the main effect size estimate directly from the dataset, the only function needed is the *convert_df()* function. Users must specify the effect measure that should be estimated (via the ‘measure’ argument), and the function will automatically estimate a main effect size, standard error, and 95% CI from any relevant input data stored in the dataset.

**Figure:**
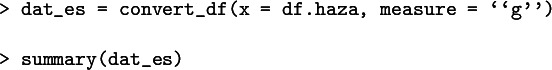

#### 3.2.2 Flexibility in input data

We describe this *convert_df()* function as flexible because—even if a dataset contains many types of input data—this function adaptively identifies the input data required to estimate an effect size in the dataset, without requiring any further indication by the users. The output of the *convert_df()* function systematically indicates to the users which type of input data was used to estimate the effect size. In the present example, nine types of input data enabled to compute an effect size (see the ‘input data available’ column in Figure 3), and eight were actually used to estimate the main effect size (see the ‘input data used’ column in the Figure 3). This feature allows users—for example—to easily conduct further analyses exploring whether the type of input data chosen to estimate the main effect size is associated with effect size magnitude.

### 3.3 Example: generate consistency indicators for overlapping input data using R

When several overlapping types of input data (i.e., multiple statistical indicators) are available for the same estimand, users can select the procedure used to estimate a main effect size via the ‘es_selected’ argument. As a general rule, we recommend users to either select the pre-defined hierarchies (‘es_selected = “auto”) or define manually the hierarchy adapted to their situation (using the ‘es_selected = “hierarchy”). In this example, we manually indicate preferring estimating the Hedges’ g in priority from the means (plus either the standard deviation, standard error, or confidence interval), then from Cohen’s d values, then from *t*-test and ANOVA values, then from eta-square values. These input data have been chosen for this example, but many more types of input data can be used to estimate an SMD (see https://metaconvert.org/input.html).

**Figure:**
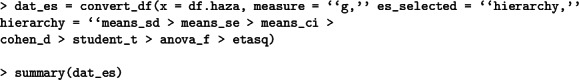

For each row with overlapping input data, the *convert_df()* function outputs the statistical indicators of the consistency of effect sizes produced by different types of input data. Relying on this output, it becomes very easy for users to have objective indicators of the consistency of effect sizes (using the % of overlap between 95% CIs, or the SD of the effect size values) as well as to make subjective judgments by plotting the results generated by the *convert_df()* function (Figure 4).

### 3.4 Example: aggregate dependent effect sizes using R

If a meta-analytic dataset contains dependent effect sizes, the *aggregate_df()* function enables it to summarize, for each clustering unit, all dependent effect sizes into one independent effect size. Three formulas are available.^13^ ^,^ ^20^ The first synthesizes dependent effect sizes coming from the same participants and is appropriate when studies contain multiple effect sizes due to the completion of several outcome measures by the same participants. This formula can be applied by using the ‘dependence = “outcomes”’ argument. The second synthesizes dependent effect sizes coming from the same participants and is appropriate when studies contain multiple effect sizes due to the inclusion of the same participants but at different time-points. This formula can be applied by using the ‘dependence = “times”’ argument. The third synthesizes dependent effect sizes coming from different participants and is appropriate when studies contain multiple effect sizes due to the presence of multiple subgroups (e.g., a given study reports two effect sizes, one per sex of the participant). This formula can be applied by using the ‘dependence = “subgroups”’ argument. The name of the column of the dataset containing information on the clustering unit is indicated using the ‘agg_fact’ argument.

**Figure:**
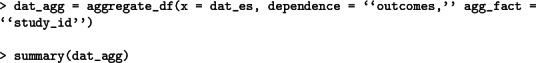

### 3.5 Example: compare datasets using R

Last, because comparing datasets created by independent pairs of data extractors can be an overwhelming task, the *compare_df()* function allows us to easily visualize differences between two datasets. This function requires passing the two datasets, and indicating the desired output format (either an html document, or an excel, text or csv file). The function returns only the rows with differences between datasets, with grey values indicating consistent information, and colored information indicating differences between the two datasets. A noteworthy feature of this function is the ability of users to specify certain columns that will be employed to reorder the datasets before conducting comparisons. This process ensures that if two datasets differ in terms of their row count or sequence, the *compare_df()* function will detect this discrepancy and automatically align the datasets based on the identification of unique values within these specified columns (see Figure 4).

**Figure:**
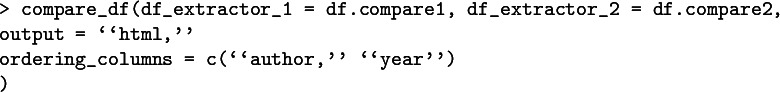

## 4 Discussion

Estimating an effect size is a crucial aspect of conveying the findings of scientific research in the sciences. This manuscript presents the R package metaConvert, which provides convenient access to a large number of formulas designed to estimate and convert between 11 effect size measurements. A web application with GUI interface complements the R package, facilitating access to the key functions without mastering the R language.

It may be argued that the approach we have developed for the handling of overlapping types of input data has direct implications for the design of certain forms of evidence synthesis. For example, an increasing number of works are striving to synthesize all data within a research field.^21^ ^–^ ^23^ In particular, authors have proposed a novel form of evidence synthesis, namely MARDs (meta-analytical research domains),^24^ with the aim of extracting and making accessible the information from all RCTs (randomized controlled trials) within a given field. One of the key objectives of MARDs is to provide a unified dataset for all future meta-analysts, thereby eliminating the need for them to re-extract data from RCTs that have already been identified in a MARD. This approach would promote consistency of results across independent meta-analyses, which has been pointed out as a major concern in many fields.^25^ ^,^ ^26^ However, if a MARD makes available only one type of input data per estimand (e.g., non-adjusted post-test means) while others are available (such as pre-test means, crude, or adjusted mean changes), this can give rise to exhaustiveness issues. It may therefore be advantageous to implement a system analogous to that proposed in metaConvert in MARDs, which would provide users with access to all types of input data available for a given estimand. This approach can also be advantageous in the context of umbrella reviews, which aim to synthesize systematic reviews and meta-analyses within a specific field.^27^ Variations in the selection of input data between meta-analyses could indeed be a potential source of inconsistency in overlapping meta-analyses.^15^ ^,^ ^28^

The metaConvert tools have several notable strengths. First, the automatic and flexible process of the metaConvert tools is a direct response to the needs expressed by researchers carrying out meta-analyses,^18^ and enables researchers in many scientific fields to rely on this tool to easily estimate different effect size measures from a wide range of (potentially interdependent) input data without the risk of making calculation errors. Second, our innovative system addressing situations with overlapping input data represents the first concrete effort to mitigate the risk of cherry-picking when meta-analysts are faced with a choice between several types of input data to estimate the same effect. Last, because the metaConvert tools are freely accessible, they align with open science principles and facilitate its adoption by a broad community.

However, this suite comes with limitations. First, there are currently no universal methods or criteria to assess the consistency of effect sizes generated by overlapping input data. Given the growing interest in this issue,^15^ it is possible that new methods/criteria emerge in the future. Rapid implementation of these new methods in the metaConvert tools will be a priority. Second, while our tools make it possible to estimate 11 effect measures, many more effect measures were developed in the literature.^29^ In concertation with the users of our tools, we aim to make other effect size measures progressively accessible. Third, although we tried to be comprehensive in the synthesis of the formulas used to estimate/convert the 11 effect measures available in our tools, for feasibility reasons, we were not able to include all the formulas available in the literature (e.g., while our tool proposed six approaches to convert OR to RR, other approaches have been proposed^30^).

Overall, the metaConvert tools will enhance end users’ ability to estimate effect sizes from various types of input data. Future developments of this suite—based on the needs expressed by our users and the community^18^—are planned to continue offering a tool that can be used at no cost by a very wide audience.

## Supporting information

Gosling et al. supplementary material Gosling et al. supplementary material

## Data Availability

The data that support the findings of this study are openly available on Github at https://github.com/CorentinJGosling/metaconvert and https://github.com/cran/metaConvert.

## Note

i. This feature has the potential to be beneficial in two distinct scenarios. First, in instances where disparate types of input data are indicative of the same estimand (e.g., a study reporting the means and standard deviations, a t-test value, and a t-test p-value), this feature enables users to readily identify the presence of errors. Indeed, in this situation, discrepancies in effect size estimates generated from different types of input data can be attributed to errors or approximations. These errors may originate from the meta-analyst team (during data extraction) or from the authors of the primary studies (during the reporting of results). Second, when different types of input data are indicative of slightly different estimands (such as two risk ratios and 95% confidence intervals, one of which is crude and the other adjusted for the effects of some covariates; or two standardized mean differences, one obtained from post-test means and one from mean change), this feature allows users to readily assess the consistency of effect sizes generated from different types of input data, and thus the impact on the data analysis. Indeed, when a meta-analyst elects to combine effect sizes that are indicative of slightly different estimands, it is currently standard practice to extract a single type of data (e.g., the risk ratio from the maximally adjusted model; or only the post-test means to generate a standardized mean difference) and then to investigate whether studies that utilize different types of input data produce effect sizes of disparate magnitude through the use of meta-regression. However, this strategy is susceptible to bias due to the potential for confounding factors to differ between studies reporting disparate types of information. In contrast, the strategy employed in metaConvert ensures the generation of a consistency indicator within the same studies. The impact of the types of input data on effect size magnitude is thus examined while holding all other variables constant.

## Supplementary material

To view supplementary material for this article, please visit https://github.com/CorentinJGosling/metaconvert.
