# Supplementary material for: metaConvert: an automatic suite for estimation of 11 different effect size measures and flexible conversion across them
Source: Res Synth Methods. 2025 Apr 2;16(3):575–86. doi: 10.1017/rsm.2025.11 (PMC12527507; doi:10.1017/rsm.2025.11)
Supplement: Gosling et al. supplementary material [file S1759287925000110sup001.pdf]

# Supplementary Materials

## *'metaConvert: an automatic suite for estimation of 11 different effect size measures and flexible conversion across them'*

Corentin J. Gosling*, PhD, Samuele Cortese, MD, PhD, Marco Solmi, MD, PhD, Belen Haza, PhD, Eduard Vieta, MD, PhD, Richard Delorme, MD, PhD, Paolo Fusar-Poli[Φ], MD, PhD & Joaquim Radua[Φ], MD, PhD

*Correspondence concerning this article should be addressed to: Corentin J. Gosling, 200 Avenue de la République, Paris Nanterre University, 92000, Nanterre, France. Email: cgosling@parisnanterre.fr

# Supplementary Figure 1.

## Comparison of the features proposed in **metaConvert** with those proposed in other existing software programs

| | metaConvert | esc | compute.es | esci | metafor | RevMan | CMA |
|---|---|---|---|---|---|---|---|
| Automatic workflow | ✓✓✓ | ✗ | ✗ | ✗ | ✓ | ✗ | ✓✓ |
| Number of effect measures | ✓✓ | ✓ | ✓ | ✓ | ✓✓✓ | ✓✓ | ✓✓ |
| Conversion between effect measures | ✓✓✓ | ✓✓ | ✓✓ | ✓ | ✓✓ | ✓ | ✓✓ |
| Aggregation of ES in multilevel situations | ✓ | ✗ | ✗ | ✗ | ✓✓✓ | ✗ | ✓ |
| Multiple ES estimations per comparison | ✓✓✓ | ✗ | ✗ | ✗ | ✗ | ✗ | ✗ |
| Dataset comparison | ✓✓✓ | ✗ | ✗ | ✗ | ✗ | ✗ | ✗ |
| Meta-analytic calculations | ✗ | ✗ | ✗ | ✓ | ✓✓✓ | ✓✓ | ✓✓ |
| Graphical-User Interface (no coding required) | 🖱 | </> | </> | 🖱 | </> | 🖱 | 🖱 |
| Price | $ | $ | $ | $ | $ | $ | $$$ |

## Supplementary Figures 2.

*Tutorial for the metaConvert web-app [https://metaconvert.org/]*

# Tutorial 1

*Automatic computation of effect sizes from a well-formatted dataset.*

# Supplementary Figures 2.

*Tutorial for the metaConvert web-app [https://metaconvert.org/]*

**Formatting dataset (1/5)** Loading dataset Options Calculation Interpretation

Step 1. Go to the « Input data » tab of the metaConvert website .

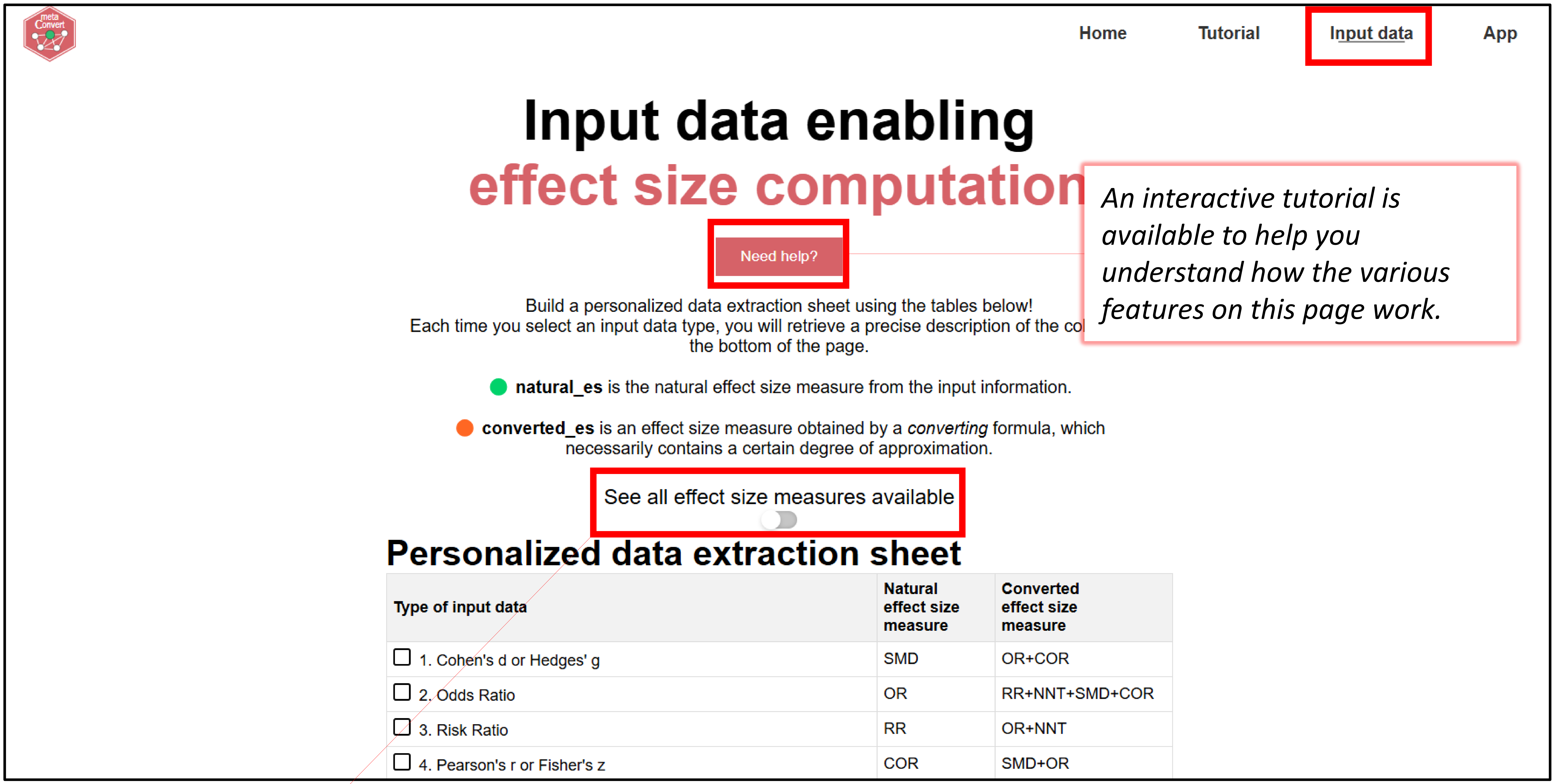


*By clicking on this switch button, you can access the list of effect size measures available and their description*

# Supplementary Figures 2.

*Tutorial for the metaConvert web-app [https://metaconvert.org/]*

**Formatting dataset (2/5)** Loading dataset Options Calculation Interpretation

## Step 2. Generate appropriate tables

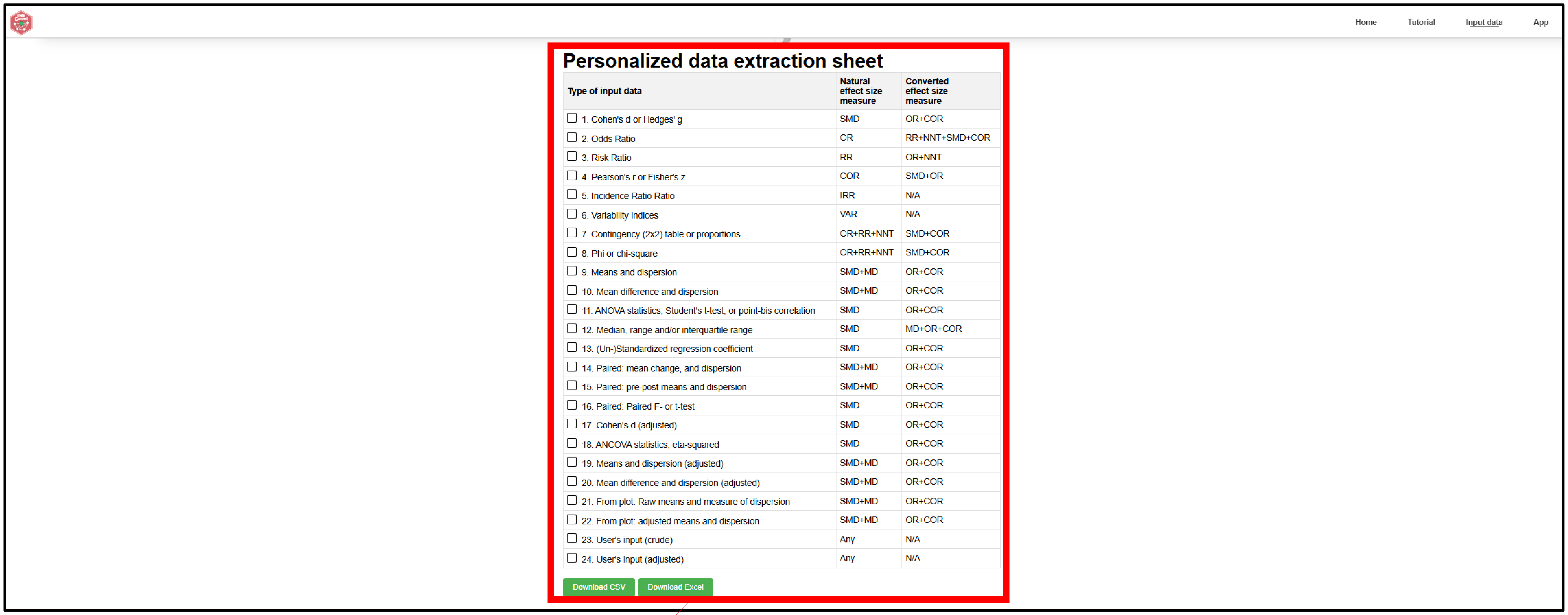


**Personalized data extraction sheet**

| Type of input data | Natural effect size measure | Converted effect size measure |
|---|---|---|
| ☐ 1. Cohen's d or Hedges' g | SMD | OR+COR |
| ☐ 2. Odds Ratio | OR | RR+NNT+SMD+COR |
| ☐ 3. Risk Ratio | RR | OR+NNT |
| ☐ 4. Pearson's r or Fisher's z | COR | SMD+OR |
| ☐ 5. Incidence Ratio Ratio | IRR | N/A |
| ☐ 6. Variability indices | VAR | N/A |
| ☐ 7. Contingency (2x2) table or proportions | OR+RR+NNT | SMD+COR |
| ☐ 8. Phi or chi-square | OR+RR+NNT | SMD+COR |
| ☐ 9. Means and dispersion | SMD+MD | OR+COR |
| ☐ 10. Mean difference and dispersion | SMD+MD | OR+COR |
| ☐ 11. ANOVA statistics, Student's t-test, or point-bis correlation | SMD | OR+COR |
| ☐ 12. Median, range and/or interquartile range | SMD | MD+OR+COR |
| ☐ 13. (Un-)Standardized regression coefficient | SMD | OR+COR |
| ☐ 14. Paired: mean change, and dispersion | SMD+MD | OR+COR |
| ☐ 15. Paired: pre-post means and dispersion | SMD+MD | OR+COR |
| ☐ 16. Paired: Paired F- or t-test | SMD | OR+COR |
| ☐ 17. Cohen's d (adjusted) | SMD | OR+COR |
| ☐ 18. ANCOVA statistics, eta-squared | SMD | OR+COR |
| ☐ 19. Means and dispersion (adjusted) | SMD+MD | OR+COR |
| ☐ 20. Mean difference and dispersion (adjusted) | SMD+MD | OR+COR |
| ☐ 21. From plot: Raw means and measure of dispersion | SMD+MD | OR+COR |
| ☐ 22. From plot: adjusted means and dispersion | SMD+MD | OR+COR |
| ☐ 23. User's input (crude) | Any | N/A |
| ☐ 24. User's input (adjusted) | Any | N/A |

Download CSV Download Excel

*The summary table allows you :*

1) *to select the type of input data you want to appear in your data extraction sheet*
2) *to automatically generate tables (below) that gives you a lot of information on the format required*

# Supplementary Figures 2.

*Tutorial for the metaConvert web-app [https://metaconvert.org/]*

**Formatting dataset (3/5)** Loading dataset Options Calculation Interpretation

## Step 3. Interpretation of the generated tables

*Name of input data*

### 11. ANOVA statistics, Student's t-test, or point-bis correlation

.xlsx .txt

*Fictitious well-formatted datasets for this type of input data allowing you to have examples on which they can rely*

| natural_es | converted_es | anova_f | anova_f_pval | student_t | student_t_pval | pt_bis_r | pt_bis_r_pval | etasq | n_exp | n_nexp |
|---|---|---|---|---|---|---|---|---|---|---|
| D+G | OR+R+Z | ✓ | × | × | × | × | × | × | ✓ | ✓ |
| D+G | OR+R+Z | × | ✓ | × | × | × | × | × | ✓ | ✓ |
| D+G | OR+R+Z | [illegible] | [illegible] | [illegible] | [illegible] | [illegible] | [illegible] | [illegible] | ✓ | ✓ |
| D+G | OR+R+Z | [illegible] | [illegible] | [illegible] | [illegible] | [illegible] | [illegible] | [illegible] | ✓ | ✓ |
| D+G | OR+R+Z | × | × | × | × | ✓ | × | × | ✓ | ✓ |
| D+G | OR+R+Z | × | × | × | × | × | ✓ | × | ✓ | ✓ |
| D+G | OR+R+Z | × | × | × | × | × | × | ✓ | ✓ | ✓ |

*Names of the variables that should be indicated in your dataset. These names cannot be changed. As shown in the fictitious datasets, if you have access to the value of an ANOVA F-test, it should be inserted in a column named « anova_f » to have an effect size estimated from the metaConvert tools.*

**anova_f** = F-test value from an ANOVA with a binary independent variable.
**anova_f_pval** = p-value from an ANOVA with a binary independent variable.
**student_t** = Student's t-test value.
**student_t_pval** = p-value from value from a Student's t-test value.
**pt_bis_r** = correlation coefficient value from a point-biserial correlation.
**pt_bis_r_pval** = p-value of a point-biserial correlation.
**etasq** = Eta-squared value from an ANOVA with a binary independent variable.
**n_exp & n_nexp** = number of participants in the exposed and non-exposed groups, respectively.

# Supplementary Figures 2.

*Tutorial for the metaConvert web-app [https://metaconvert.org/]*

**Formatting dataset (4/5)** Loading dataset Options Calculation Interpretation

## Step 3. Interpretation of the generated tables

*Each row of the table presents a minimal combination of input data allowing to estimate/convert the effect size measures listed in the two left columns*

✓ *: means that the information is indicated in the dataset*

✗ *: means that the information is not required to generate an effect size*

ata App

### 11. ANOVA statistics, Stude

.xlsx .txt

| natural_es | converted_es | anova_f | anova_f_pval | student_t | student_t_pval | pt_bis_r | pt_bis_r_pval | etasq | n_exp | n_nexp |
|---|---|---|---|---|---|---|---|---|---|---|
| D+G | OR+R+Z | ✓ | × | × | × | × | × | × | ✓ | ✓ |
| D+G | OR+R+Z | × | ✓ | × | × | × | × | × | ✓ | ✓ |
| D+G | OR+R+Z | × | × | ✓ | × | × | × | × | ✓ | ✓ |
| D+G | OR+R+Z | × | × | × | ✓ | × | × | × | ✓ | ✓ |
| D+G | OR+R+Z | × | × | × | × | ✓ | × | × | ✓ | ✓ |
| D+G | OR+R+Z | × | × | × | × | × | ✓ | × | ✓ | ✓ |
| D+G | OR+R+Z | × | × | × | × | × | × | ✓ | ✓ | ✓ |

*This column lists the effect size measure(s) estimated directly from the input data*

*This column lists the effect size measure(s) that can be converted from the input data*

# Supplementary Figures 2.

*Tutorial for the metaConvert web-app [https://metaconvert.org/]*

**Formatting dataset (5/5)** Loading dataset Options Calculation Interpretation

## Step 3. Interpretation of the generated tables

**11. ANOVA statistics, Student's t-test, or point-bis correlation**

.xlsx .txt

| natural_es | converted_es | anova_f | anova_f_pval | student_t | student_t_pval | pt_bis_r | pt_bis_r_pval | etasq | n_exp | n_nexp |
|---|---|---|---|---|---|---|---|---|---|---|
| D+G | OR+R+Z | ✓ | × | × | × | × | × | × | ✓ | ✓ |
| D+G | OR+R+Z | × | ✓ | × | × | × | × | × | ✓ | ✓ |
| D+G | OR+R+Z | × | × | ✓ | × | × | × | × | ✓ | ✓ |
| D+G | OR+R+Z | × | × | × | ✓ | × | × | × | ✓ | ✓ |
| D+G | OR+R+Z | × | × | × | × | ✓ | × | × | ✓ | ✓ |
| D+G | OR+R+Z | × | × | × | × | × | ✓ | × | ✓ | ✓ |
| D+G | OR+R+Z | × | × | × | × | × | × | ✓ | ✓ | ✓ |

**anova_f** = F-test value from an ANOVA with a binary independent variable.
**anova_f_pval** = p-value from an ANOVA with a binary independent variable.
**student_t** = Student's t-test value.
**student_t_pval** = p-value from value from a Student's t-test value.
**pt_bis_r** = correlation coefficient value from a point-biserial correlation.
**pt_bis_r_pval** = p-value of a point-biserial correlation.
**etasq** = Eta-squared value from an ANOVA with a binary independent variable.
**n_exp & n_nexp** = number of participants in the exposed and non-exposed groups, respectively.

*This dataset (bottom) is a direct application of the table (above).*

*Each row will lead to estimate the effect size measures listed above (SMD, OR, COR [i.e., R and Z]).*

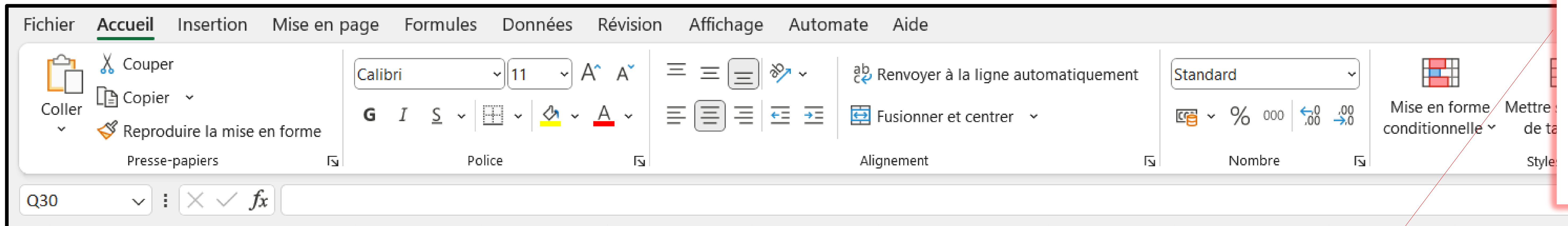

| | A | B | C | D | E | F | G | H | I | J | K |
|---|---|---|---|---|---|---|---|---|---|---|---|
| 1 | all_info_expected | study_id | anova_f | anova_f_pval | student_t | student_t_pval | pt_bis_r | pt_bis_r_pval | etasq | n_exp | n_nexp |
| 2 | anova_f | 1 | 5.94 | | | | | | | 35 | 47 |
| 3 | anova_f_pval | 2 | | 0.0058 | | | | | | 62 | 55 |
| 4 | student_t | 3 | | | 3.78 | | | | | 35 | 47 |
| 5 | student_t_pval | 4 | | | | 0.00359 | | | | 29 | 23 |
| 6 | pt_bis_r | 5 | | | | | 0.57 | | | 94 | 84 |
| 7 | pt_bis_r_pval | 6 | | | | | | 0.809 | | 85 | 90 |
| 8 | etasq | 7 | | | | | | | 0.757 | 100 | 109 |
| 9 | anova_f + anova_f_pval + etasq | 8 | 4.98 | 0.03 | | | | | 0.055 | 42 | 38 |

*This last row intends to show a dataset with overlapping input data. In this row, the metaConvert tools will estimate 3 effect sizes (one from the ANOVA F-test value, one from the ANOVA p-value, and one from the eta² value). Based on users' choice, the metaConvert will select one of these effect sizes as the main effect size and will compute consistency indicators*

# Supplementary Figures 2.

*Tutorial for the metaConvert web-app [https://metaconvert.org/]*

Formatting dataset | **Loading dataset (1/2)** | Options | Calculation | Interpretation

Once your dataset is correctly formatted, go to the App section of the website and upload it to the app.

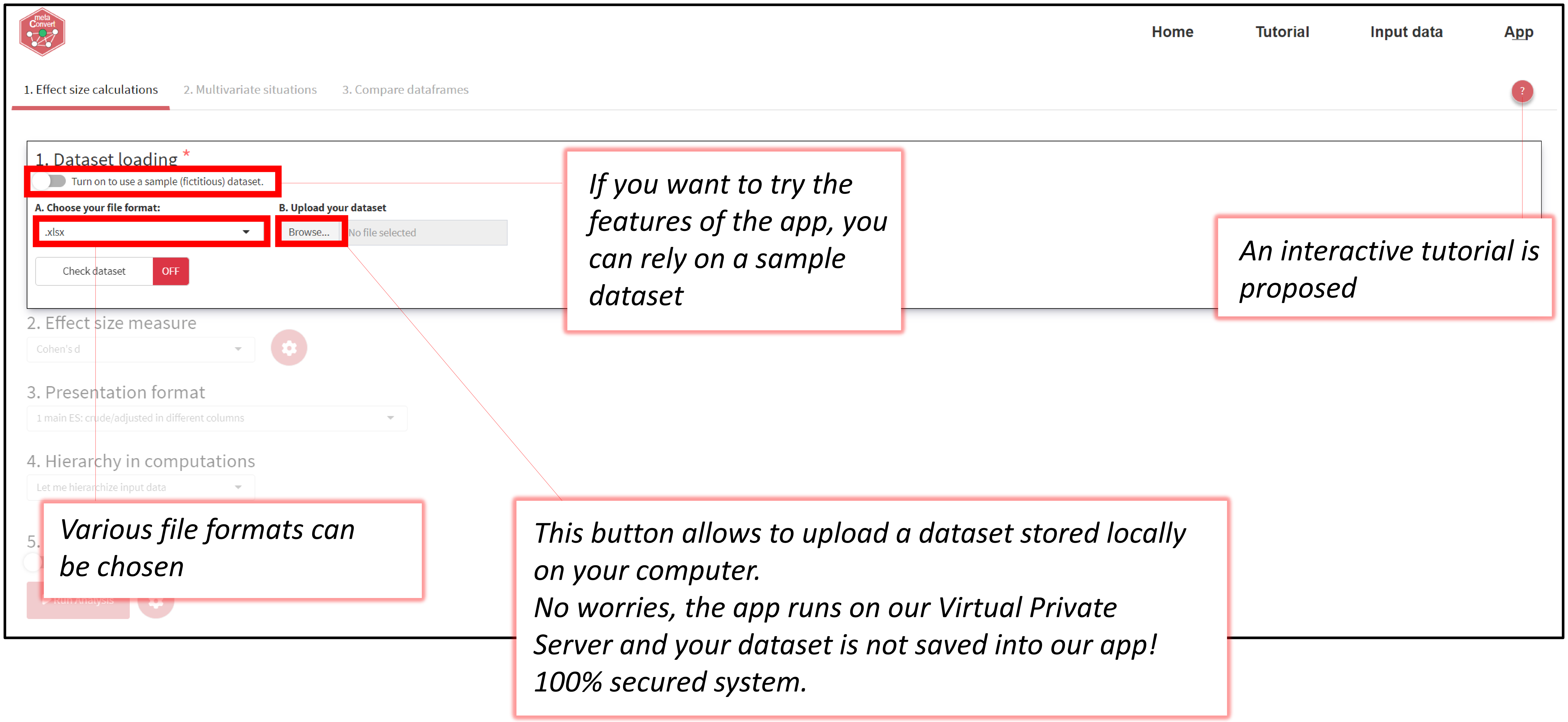

# Supplementary Figures 2.

*Tutorial for the metaConvert web-app [https://metaconvert.org/]*

Formatting dataset | **Loading dataset (2/2)** | Options | Calculation | Interpretation

Once your dataset is correctly formatted, go to the App section of the website and upload it to the app.

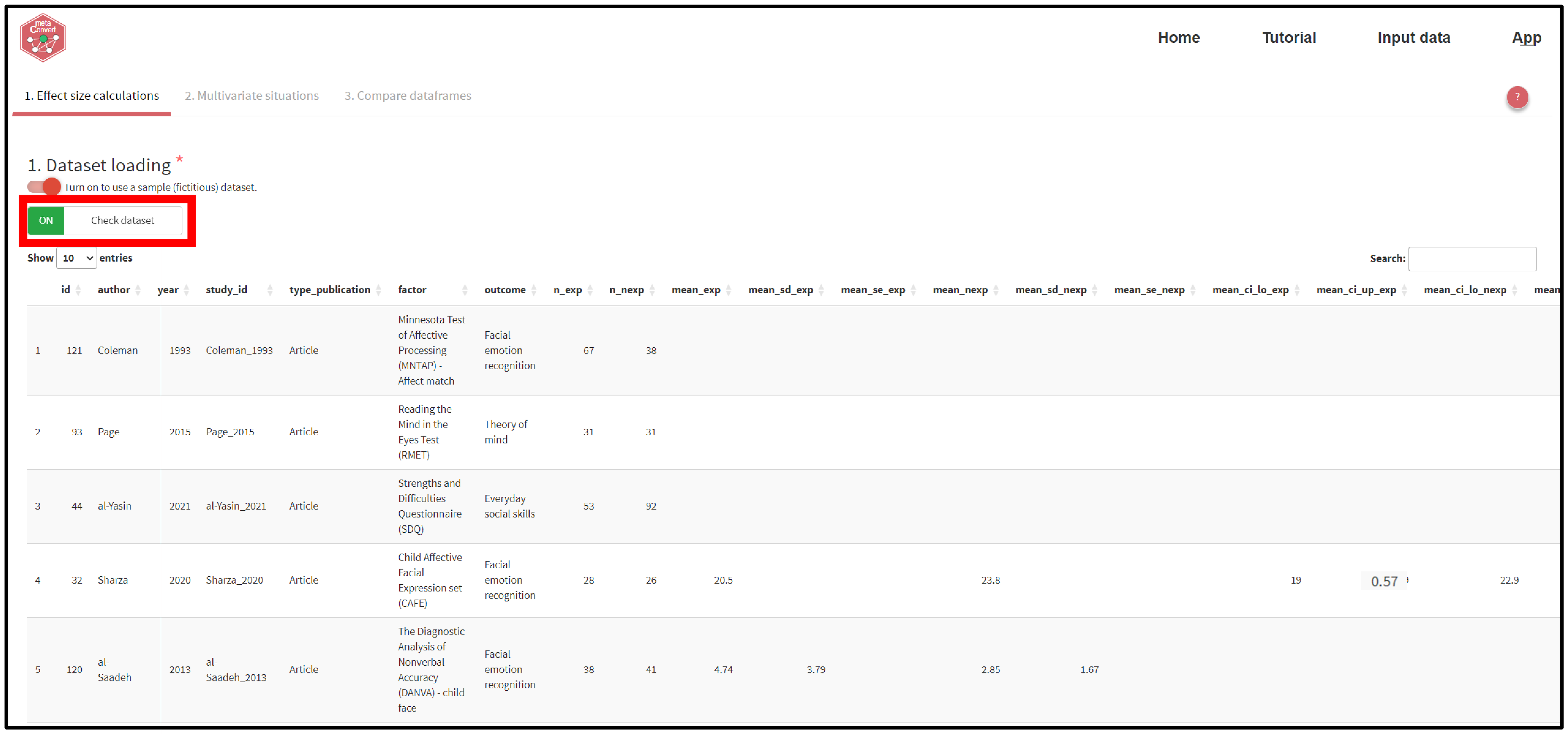


*This switch button allows you to easily visualize the uploaded dataset (and allows to check that the uploading process went well)*

# Supplementary Figures 2.

*Tutorial for the metaConvert web-app [https://metaconvert.org/]*

Formatting dataset | Loading dataset | **Options (1/1)** | Calculation | Interpretation

Once your dataset is correctly formatted, go to the App section of the website and upload it to the app.

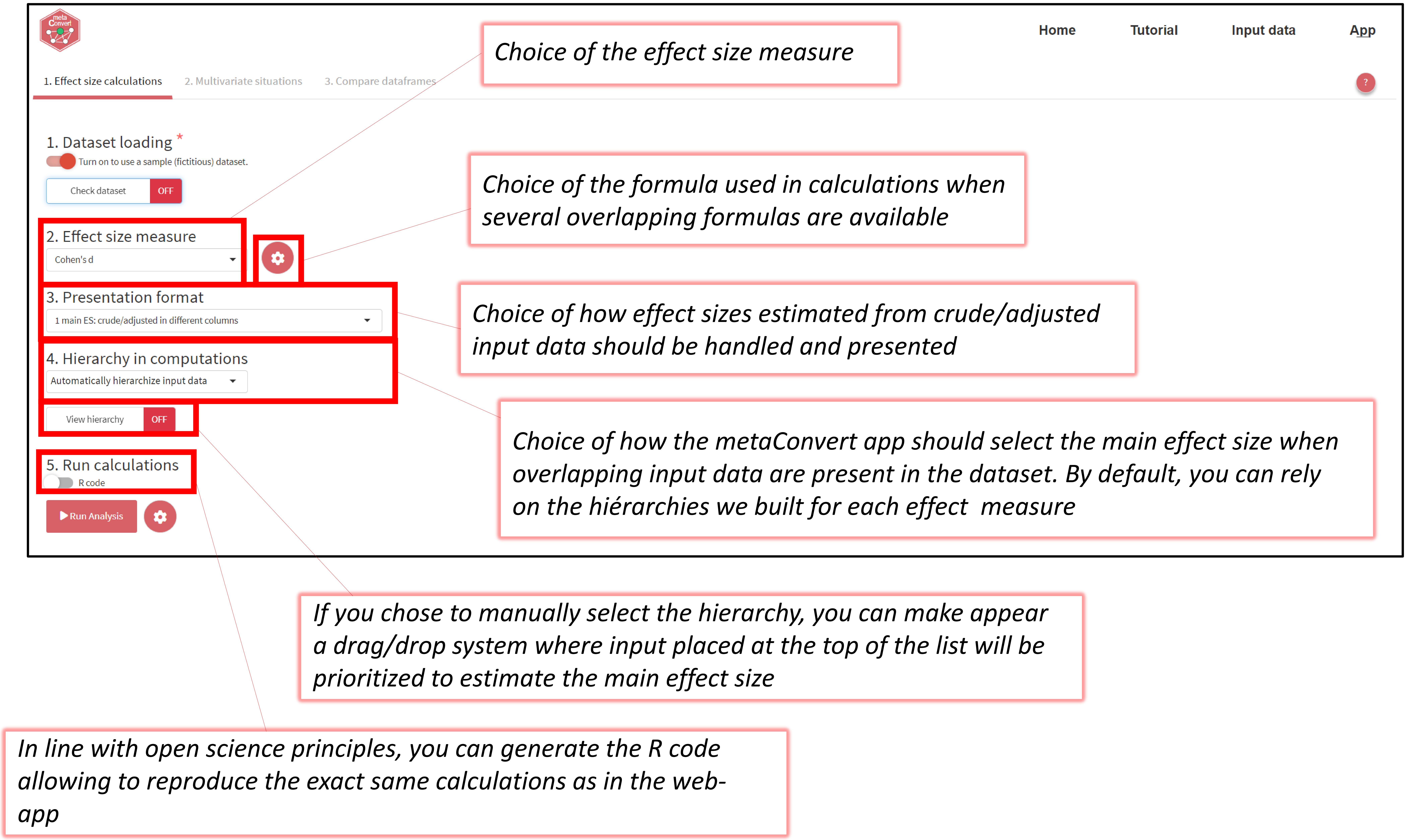

# Supplementary Figures 2.

*Tutorial for the metaConvert web-app [https://metaconvert.org/]*

Formatting dataset Loading dataset Options **Calculation (1/1)** Interpretation

When all appropriate options have been selected, calculations can be running!

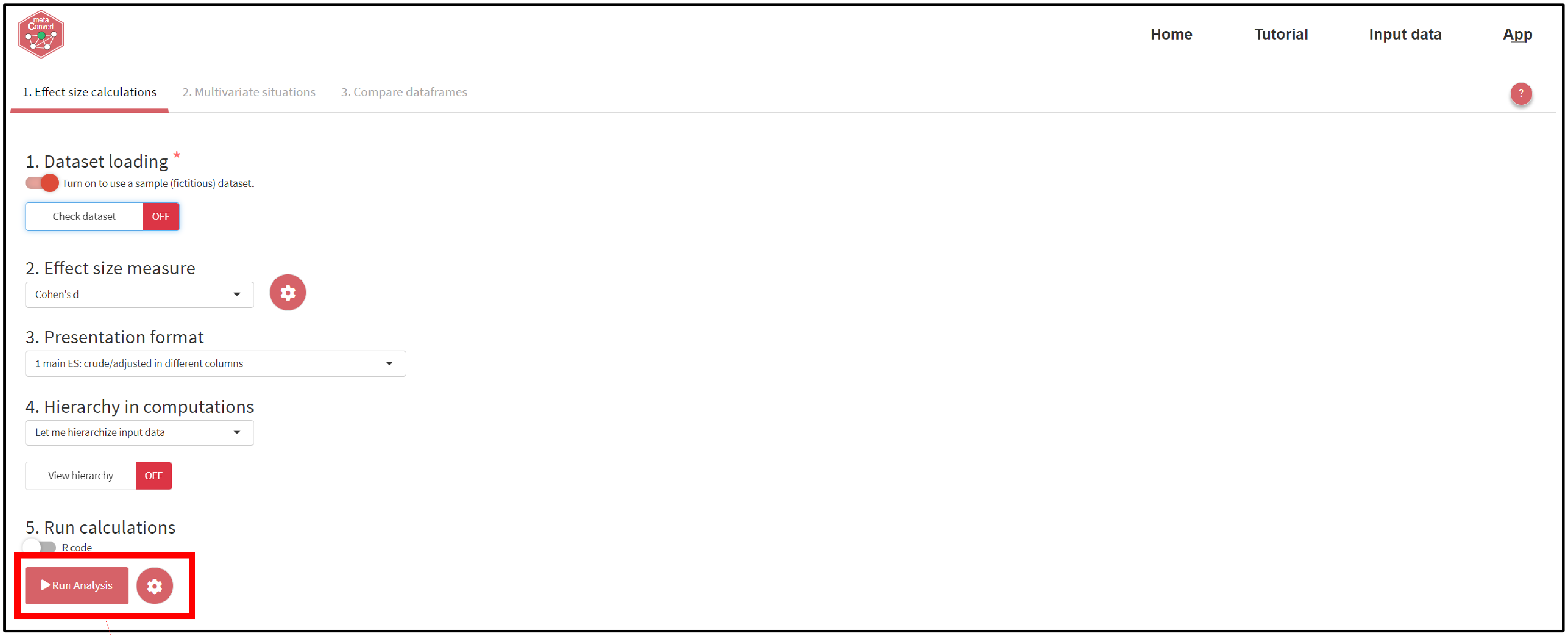


*Simply click on this button, everything is done automatically!*

# Supplementary Figures 2.

*Tutorial for the metaConvert web-app [https://metaconvert.org/]*

Formatting dataset Loading dataset Options Calculation **Interpretation (1/5)**

The first tab is a table presenting all results of the calculations.

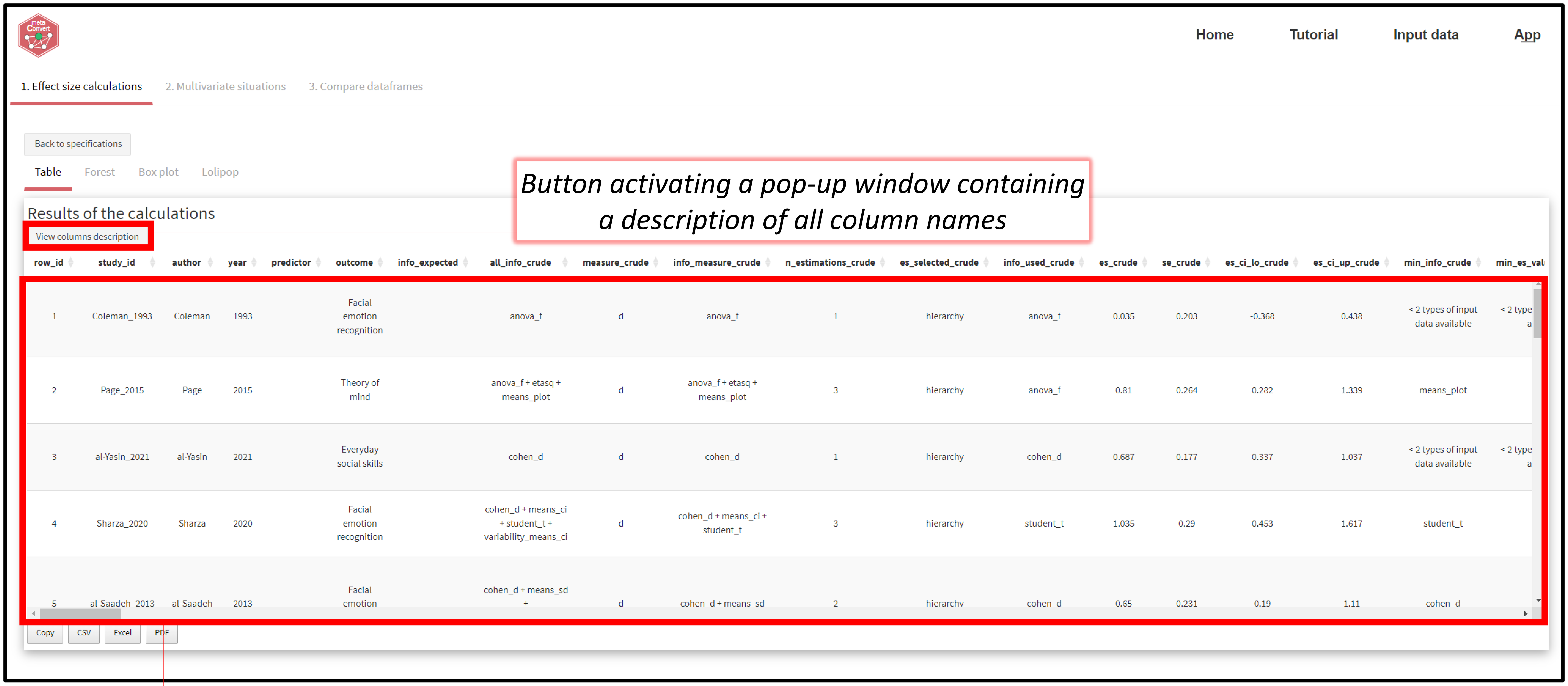


Results of the calculations

# Supplementary Figures 2.

*Tutorial for the metaConvert web-app [[https://metaconvert.org/](https://metaconvert.org/)]*

Formatting dataset Loading dataset Options Calculation **Interpretation (2/5)**

The second tab is a forest plot where the effect size values + 95% CI and input data used to estimate each effect size are presented

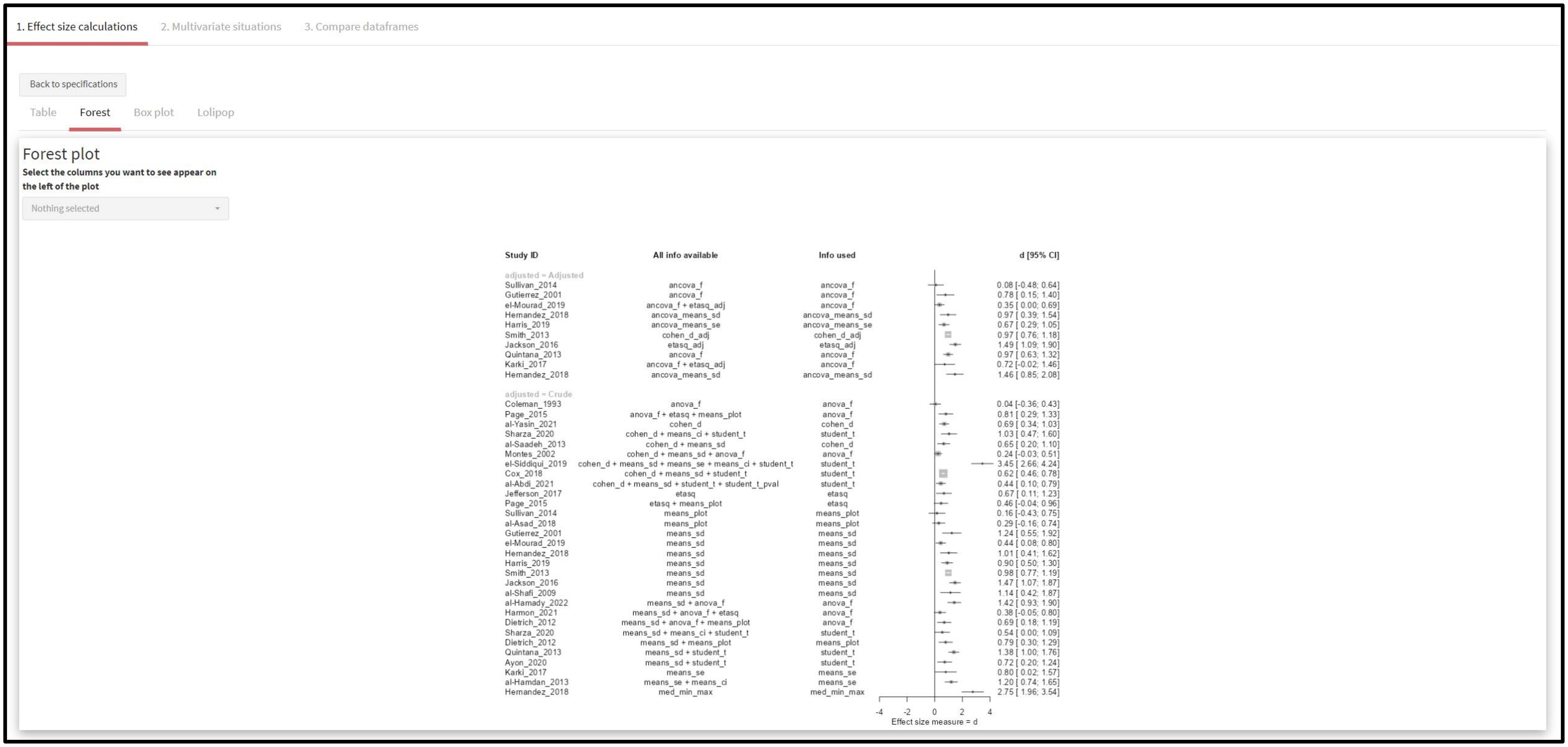

# Supplementary Figures 2.

*Tutorial for the metaConvert web-app [https://metaconvert.org/]*

Formatting dataset | Loading dataset | Options | Calculation | **Interpretation (3/5)**

The second tab is a forest plot where the effect size values + 95% CI and input data used to estimate each effect size are presented

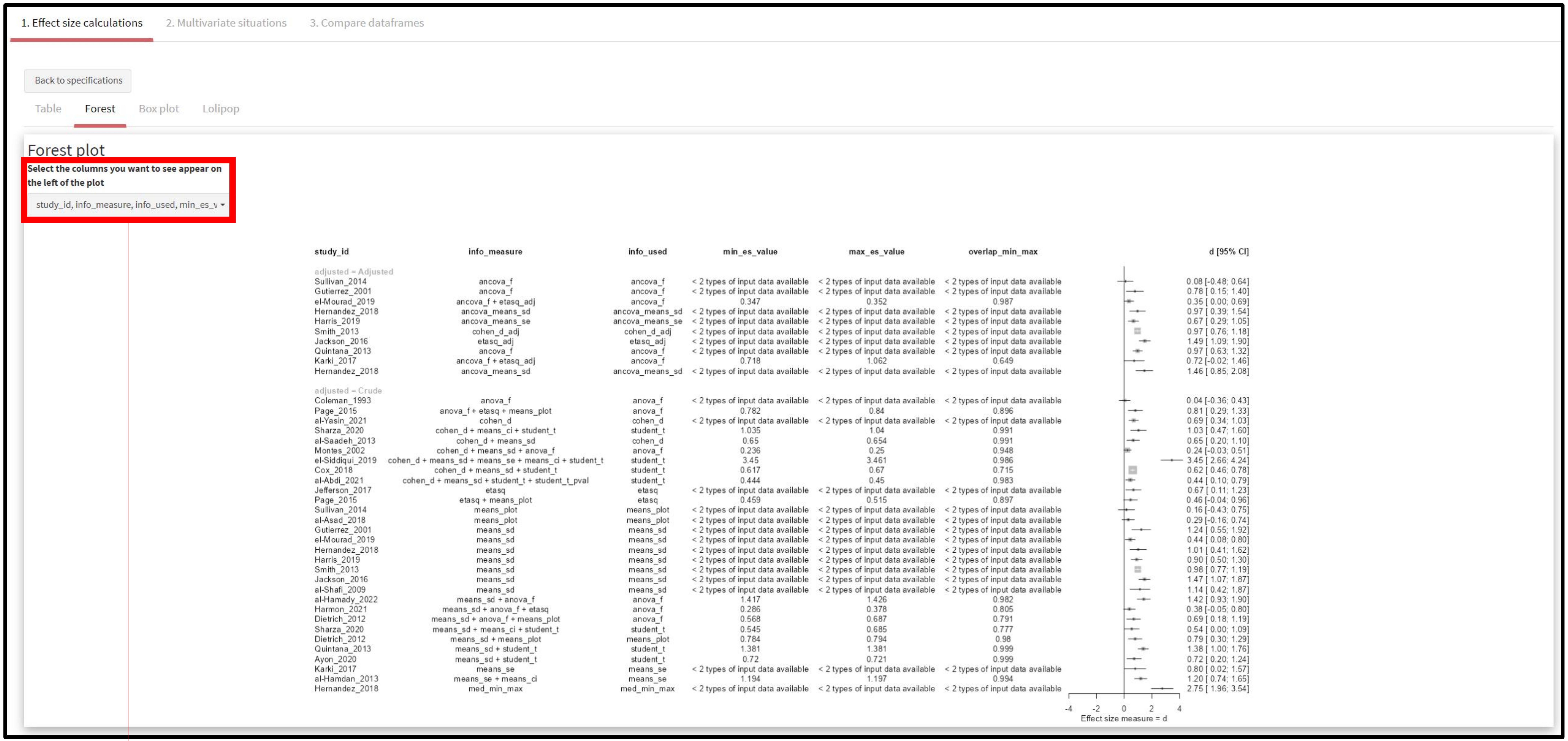


*This button allows to change the columns presented at the left of the plot. For example, here we present the minimum and maximum effect size values generated by each comparison, as well as the percentage of overlap between the 95% CIs ot these minimum and maximum effect sizes as a consistency indicator between input data types.*

*The "< 2 types of input data available" indicates that – for the current comparison – users indicate only one type of input data*

# Supplementary Figures 2.

*Tutorial for the metaConvert web-app [https://metaconvert.org/]*

Formatting dataset Loading dataset Options Calculation **Interpretation (4/5)**

The third tab is a box plot showing the effect size values depending on the input data. Outliers are highlighted in red.

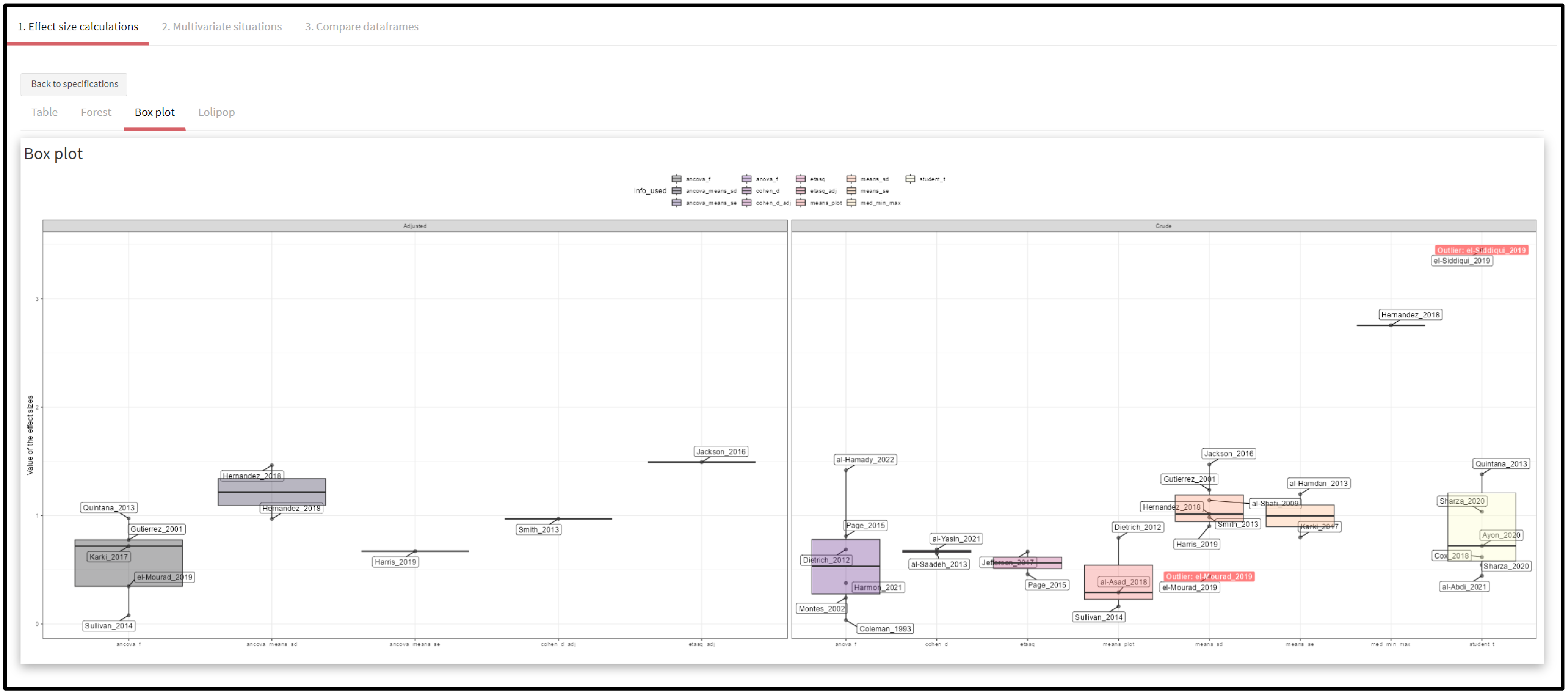

# Supplementary Figures 2.

*Tutorial for the metaConvert web-app [https://metaconvert.org/]*

Formatting dataset Loading dataset Options Calculation **Interpretation (5/5)**

The fourth tab is a lolipop plot where each comparison with more than 2 input data available are plotted
Minimum and maximum effect size values and 95% CIs are plotted in red and green, respectively.

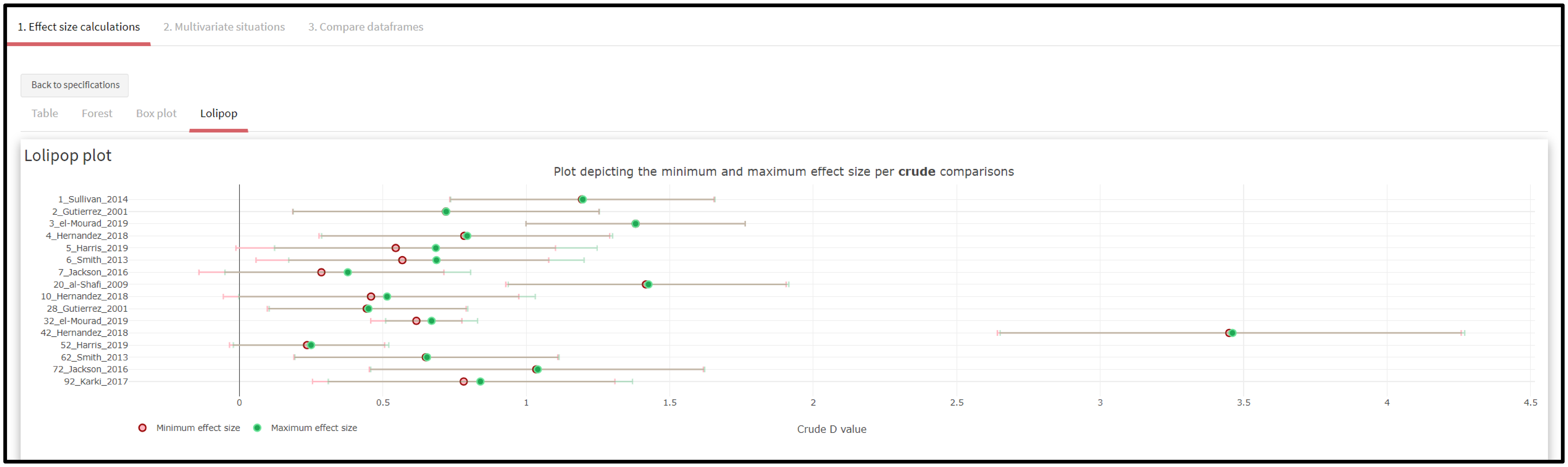

# Tutorial 2

*Aggregation of dependent effect sizes*

# Supplementary Figures 2.

*Tutorial for the metaConvert web-app [https://metaconvert.org/]*

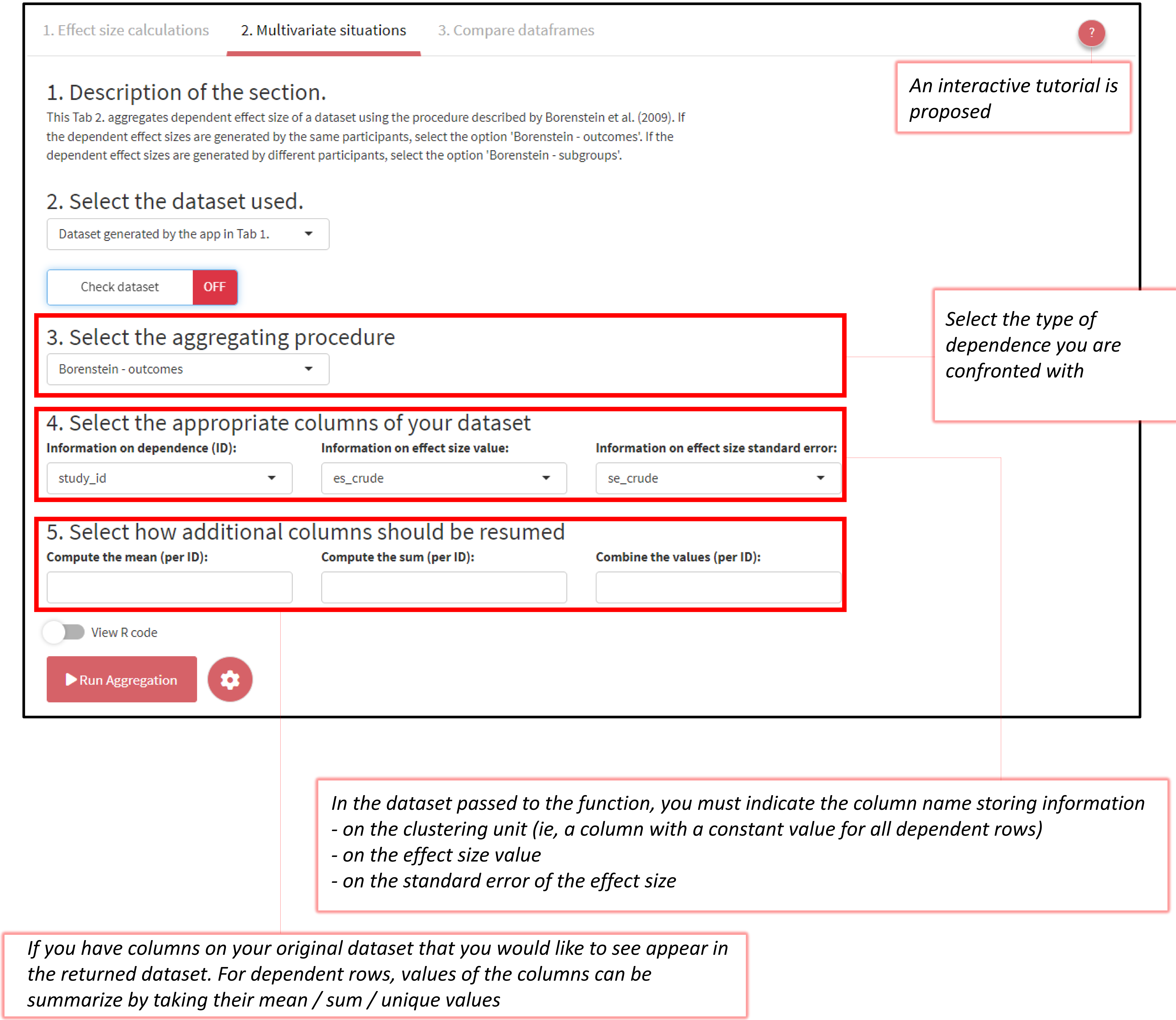

# Supplementary Figures 2.

*Tutorial for the metaConvert web-app [https://metaconvert.org/]*

1. Effect size calculations | 2. Multivariate situations | 3. Compare dataframes

?

Back to specifications

## Results of the aggregating procedure

| row_index | study_id | es | se |
|---|---|---|---|
| 1 | Coleman_1993 | 0.035 | 0.203 |
| 2 | Page_2015 | 0.6345 | 0.247134477562318 |
| 3 | al-Yasin_2021 | 0.687 | 0.177 |
| 4 | Sharza_2020 | 0.79 | 0.268959569452362 |
| 5 | al-Saadeh_2013 | 0.65 | 0.231 |
| 6 | Montes_2002 | 0.242 | 0.138 |
| 7 | el-Siddiqui_2019 | 3.45 | 0.404 |
| 8 | Cox_2018 | 0.617 | 0.081 |
| 9 | al-Abdi_2021 | 0.444 | 0.175 |
| 10 | Jefferson_2017 | 0.667 | 0.285 |
| 12 | Sullivan_2014 | 0.163 | 0.302 |
| 13 | al-Asad_2018 | 0.29 | 0.23 |
| 14 | Gutierrez_2001 | 1.237 | 0.35 |
| 15 | el-Mourad_2019 | 0.438 | 0.184 |

Copy | CSV | Excel | PDF

*The returned dataset contains, at least, the information on the clustering unit, and the aggregated effect size values and standard errors. Any additional column indicated in the Section 5 of the previous tab will be aggregated and included in the dataset.*

# Tutorial 3

*Comparisons of datasets*

# Supplementary Figures 2.

*Tutorial for the metaConvert web-app [https://metaconvert.org/]*

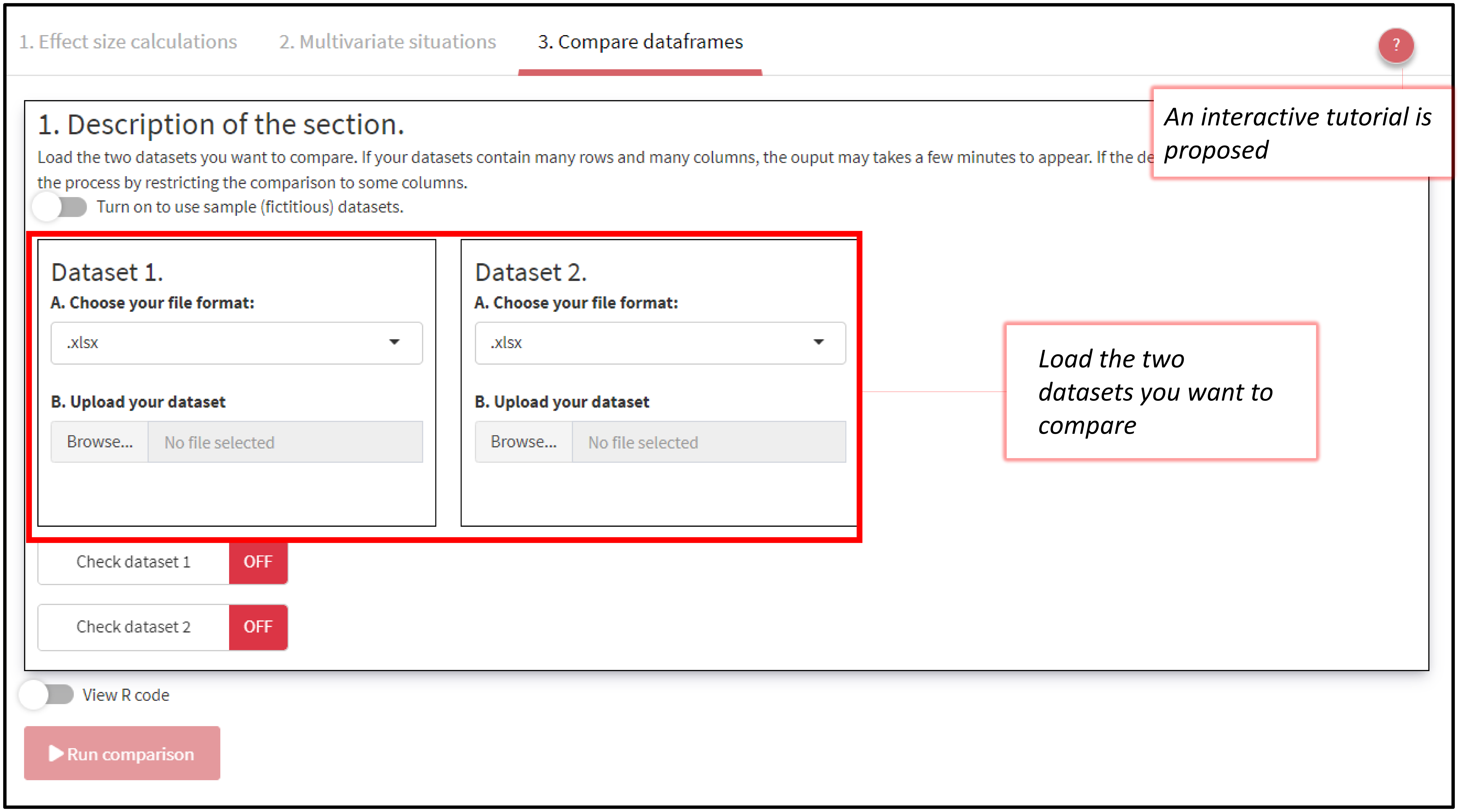

# Supplementary Figures 2.

*Tutorial for the metaConvert web-app [https://metaconvert.org/]*

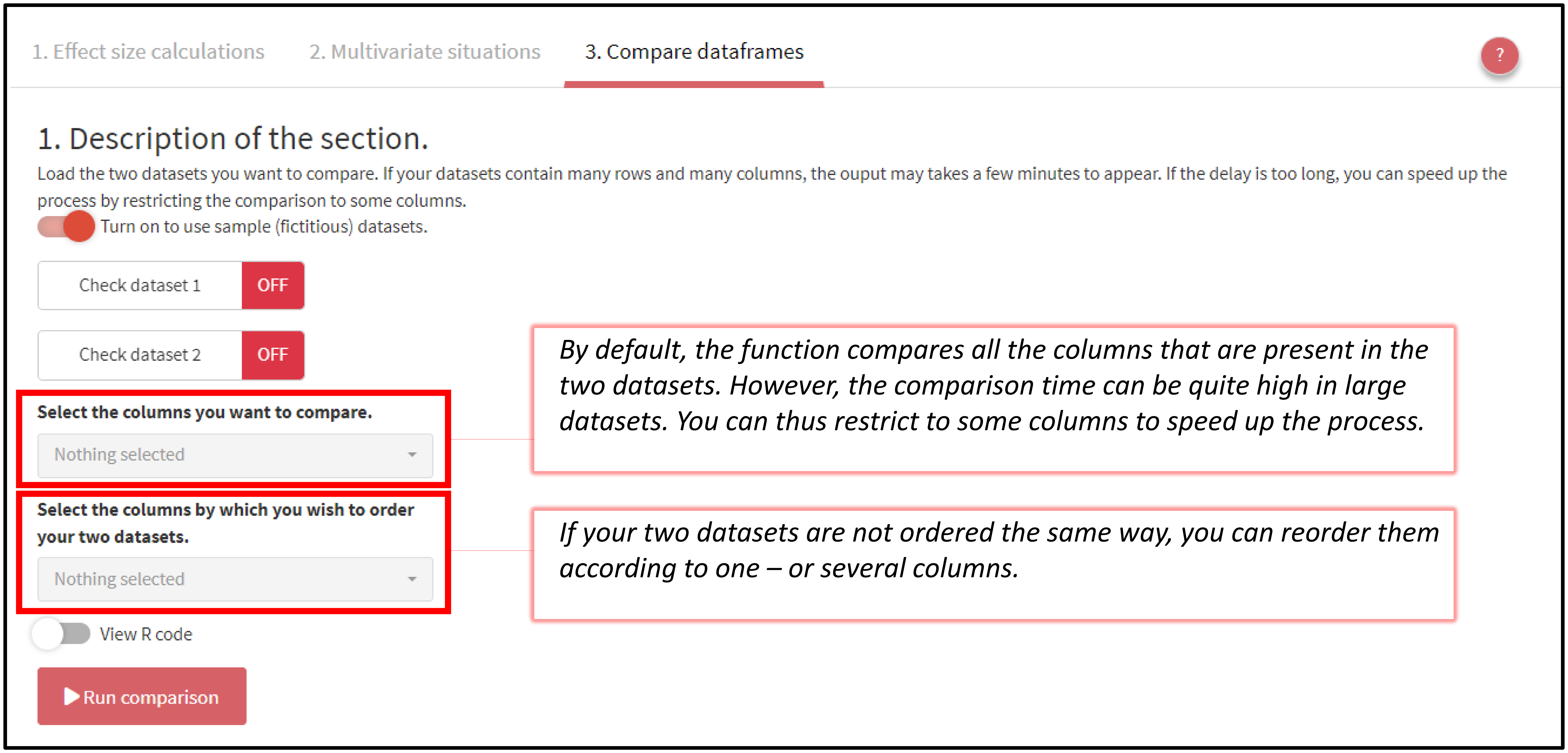

# Supplementary Figures 2.

*Tutorial for the metaConvert web-app [https://metaconvert.org/]*

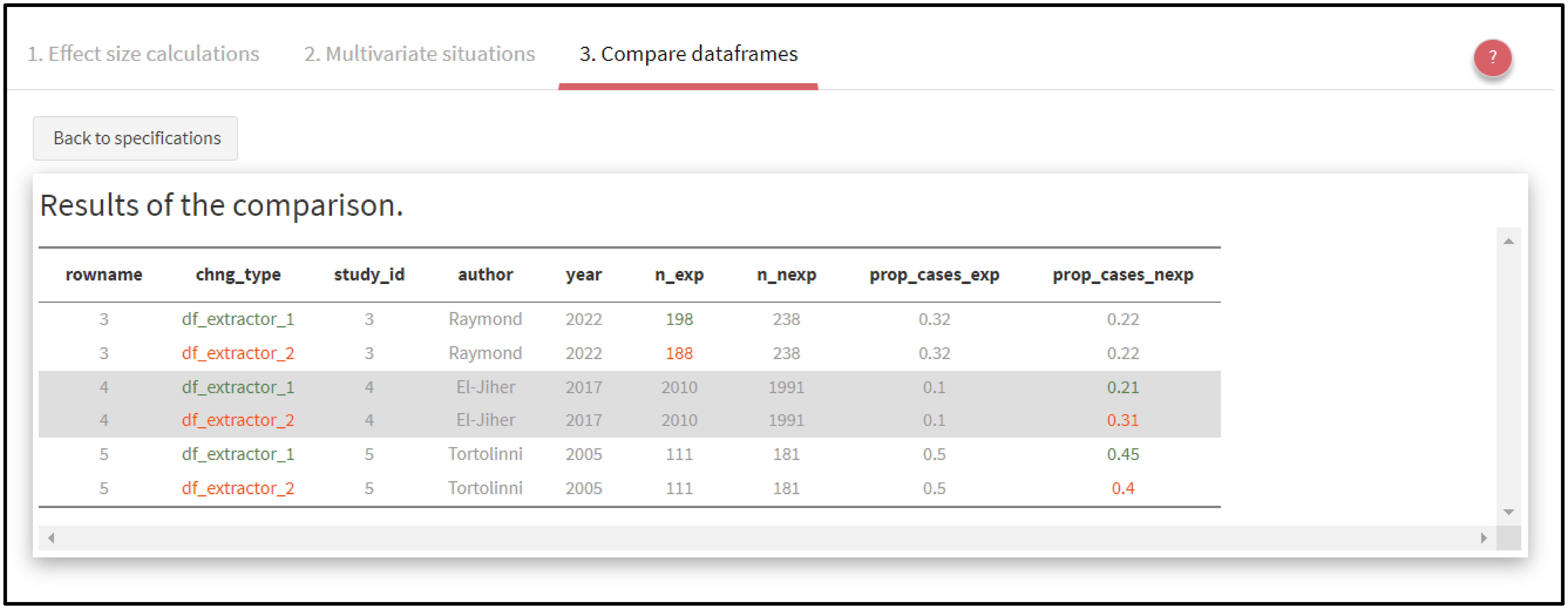


Results of the comparison.

| rowname | chng_type | study_id | author | year | n_exp | n_nexp | prop_cases_exp | prop_cases_nexp |
|---|---|---|---|---|---|---|---|---|
| 3 | df_extractor_1 | 3 | Raymond | 2022 | 198 | 238 | 0.32 | 0.22 |
| 3 | df_extractor_2 | 3 | Raymond | 2022 | 188 | 238 | 0.32 | 0.22 |
| 4 | df_extractor_1 | 4 | El-Jiher | 2017 | 2010 | 1991 | 0.1 | 0.21 |
| 4 | df_extractor_2 | 4 | El-Jiher | 2017 | 2010 | 1991 | 0.1 | 0.31 |
| 5 | df_extractor_1 | 5 | Tortolinni | 2005 | 111 | 181 | 0.5 | 0.45 |
| 5 | df_extractor_2 | 5 | Tortolinni | 2005 | 111 | 181 | 0.5 | 0.4 |

*The returned image contains only rows with at least 1 difference between the two datasets. Different values in the two datasets appear in red/green while identical values are in grey.*
